# Accelerometer-measured physical activity and sedentary time among children and their parents in the UK before and after COVID-19 lockdowns: a natural experiment

**DOI:** 10.1186/s12966-022-01290-4

**Published:** 2022-05-16

**Authors:** Ruth Salway, Charlie Foster, Frank de Vocht, Byron Tibbitts, Lydia Emm-Collison, Danielle House, Joanna G. Williams, Katie Breheny, Tom Reid, Robert Walker, Sarah Churchward, William Hollingworth, Russell Jago

**Affiliations:** 1 Centre for Exercise, Nutrition & Health Sciences, School for Policy Studies, University of Bristol, Bristol, BS8 ITZ UK; 2 Bristol Medical School, Population Health Sciences, University of Bristol, Bristol, BS8, 2PS UK; 3 Applied Research Collaboration West (NIHR ARC West), The National Institute for Health Research, University Hospitals Bristol and Weston NHS Foundation Trust, Bristol, BS1 2NT UK; 4 Communities and Public Health, Bristol City Council, Bristol, BS1 9NE UK; 5 Independent Public Member of the Project Team, Bristol, UK

**Keywords:** Physical activity, Children, COVID-19, Sedentary behaviour, Adults, SARS CoV 2

## Abstract

**Background:**

Restrictions due to the coronavirus disease 2019 (COVID-19) pandemic reduced physical activity provision for both children and their parents. Recent studies have reported decreases in physical activity levels during lockdown restrictions, but these were largely reliant on self-report methods, with data collected via unrepresentative self-report surveys. The post-pandemic impacts on children’s activity levels remain unknown. A key question is how active children become once lockdown restrictions are lifted.

**Methods:**

Active-6 is a repeated cross-sectional natural experiment. Accelerometer data from 1296 children aged 10–11 and their parents were collected in 50 schools in the Greater Bristol area, UK in March 2017-May 2018 (pre-COVID-19 comparator group), and compared to 393 children aged 10–11 and parents in 23 of the same schools, collected in May-December 2021. Mean minutes of accelerometer-measured moderate-to-vigorous physical activity (MVPA) were derived for weekdays and weekend and compared pre- and post-lockdown via linear multilevel models.

**Results:**

After adjusting for seasonality, accelerometer wear time and child/parent demographics, children’s mean weekday and weekend MVPA were 7.7 min (95% CI: 3.5 to 11.9) and 6.9 min (95% CI: 0.9 to 12.9) lower in 2021 than in 2018, respectively, while sedentary time was higher by 25.4 min (95% CI: 15.8 to 35.0) and 14.0 min (95% CI: 1.5 to 26.5). There was no evidence that differences varied by child gender or household education. There was no significant difference in parents’ MVPA or sedentary time, either on weekdays or weekends.

**Conclusions:**

Children’s MVPA was lower by 7–8 min/day in 2021 once restrictions were lifted than before the pandemic for all groups, on both weekdays and weekends. Previous research has shown that there is an undesirable age-related decline in children’s physical activity. The 8-min difference reported here would be broadly comparable to the decline that would have previously been expected to occur over a three-year period. Parents’ physical activity was similar to pre-pandemic levels. Our results suggest that despite easing of restrictions, children’s activity levels have not returned to pre-pandemic levels. There is an urgent need to understand why these changes have occurred and how long they are maintained.

**Supplementary Information:**

The online version contains supplementary material available at 10.1186/s12966-022-01290-4.

## Introduction

Physical activity is important for health among children and adults. In adulthood, physical activity is associated with reduced risk of heart disease, stroke, type 2 diabetes mellitus and many forms of cancer as well as reduced risk of depression and improved psychological well-being [1–3]. In childhood, physical activity is associated with reduced risk of obesity and improved emotional well-being [4–6]. The World Health Organization and UK Chief Medical Officers (CMO) recommend that children and young people should engage in an average of an hour of moderate-to-vigorous intensity physical activity (MVPA) per day, accumulated across the day, and that all adults should engage in 150 min or more of moderate intensity physical activity per week [3, 7, 8]. However only 41% of children aged 10–11 years in the UK meet this recommendation[9], and evidence shows a decline in physical activity throughout childhood and adolescence [9, 10], with average weekday MVPA declining at a rate of 2.2 min per year between ages 6 and 11.

The coronavirus disease 2019 (COVID-19) pandemic, and resulting restrictions to limit the spread of the virus, has had marked impacts on all aspects of society, including limiting both access and ways in which adults and children are active [11]. In England, an initial lockdown began on 23^rd^ March 2020 with the closure of non-essential businesses, hospitality, leisure facilities and playgrounds, and schools were open only to children of key workers and to vulnerable children, followed by a phased release from 13^th^ May 2020. A second short lockdown, with non-essential business and leisure facilities closed but with schools remaining open to all pupils, was in place between 5th and 24^th^ November 2020, and a third full lockdown with schools closed again to many pupils from 6^th^ January to 3^rd^ March 2021[12]. Thus, the COVID-19 pandemic has resulted in changes to physical activity opportunity via restrictions on physical education, travel to school and work, sport and outdoor play for both children and adults, and potentially changes to screen-viewing habits via home-working and online learning. Moreover, some behaviours continue even when restrictions are lifted, with some adults for example choosing to continue working from home, and many children have been out of school for varying periods of time, including isolation due to COVID-19 infection or contact.

The majority of previous studies examining physical activity during the pandemic have used convenience samples recruited via social media, retrospective measures of pre-COVID-19 activity and subjective self-report measures of physical activity [13, 14]. Most studies report large decreases in the duration and frequency of physical activity across multiple countries in both children and adults [13, 14]. Studies that have used accelerometer assessments of physical activity in healthy primary-aged children are limited with small sample sizes, but have reported decreases of 10–17 min of MVPA during and shortly after school closures, compared to pre-pandemic, in several countries [15–17]. In England, the national Active Lives Children and Young People Survey (ALCYPS) [18] found a 2.2 percentage point change in the proportion of children meeting the CMO guidelines between the summer term 2019 and 2020, with no further change between 2020 and 2021[18]. Evidence also suggests an increase in screen-viewing of 0.5 h-1.5 h per week [15, 16, 19], although some of this can be attributed to the increase in online schoolwork. Among adults, there were large changes in activity between May 2020 and May 2021, with an estimated 0.8 million fewer adults meeting the CMO guidelines in England since the pandemic began [20, 21].

The main determinants of children’s physical activity during the pandemic were found to be factors associated with the outdoor environment (such as urban area, lack of outdoor space), age, gender and socioeconomic background [14]. In England, boys aged 9–11 experienced the largest decreases in activity [18], although longitudinal changes in meeting CMO guidelines were not found to be associated with ethnicity or sex [22]. While children from the least affluent families did not have reductions in physical activity, the large socioeconomic gap in activity levels remains. Among those aged 35–54, who are more likely to be parents [23], the percentage meeting guidelines fell from 66 to 64%, with larger decreases among men but with women more likely to remain consistently less active, even during periods when restrictions were eased [20, 24]. There is a clear disparity in the impact of COVID-19 restrictions on overall activity levels for different genders and socioeconomic position.

Due to timescales, most of the evidence to date has understandably been on the immediate impact of COVID-19 restrictions on physical activity. However, it is now possible to look at longer-term impacts and a key question is how active children are once lockdown restrictions have been removed. It is crucial that we understand how children’s physical activity has been impacted beyond the immediate lockdown restrictions as early as possible, as well as the factors associated with changes, if we are to develop strategies to compensate or encourage physical activity. This paper compares accelerometer measures in children aged 10–11 from before the pandemic (2018–19) with data on children aged 10–11 in the same schools collected in May-December 2021, to determine whether reductions in physical activity remained after restrictions have eased, to characterise post-pandemic physical activity levels and whether any differences are associated with gender and socioeconomic position (SEP).

## Methods

**Fig. 1:**
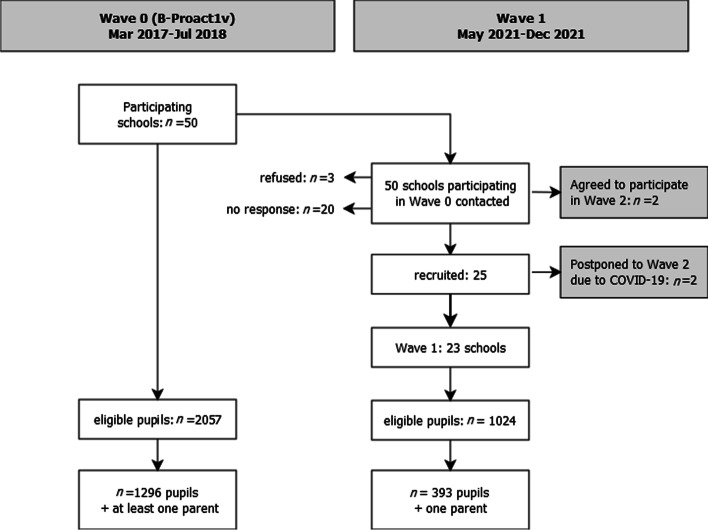
Flow diagram of participants. Note that Wave 2 data collection is still ongoing

Active-6 [25] is a repeated cross-sectional natural experiment, which compares data in a pre-COVID-19 comparator group to new data collected at two time periods after many restrictions due to the COVID-19 pandemic have lifted. This paper reports cross-sectional data from the pre-COVID-19 comparator group (which we will refer to as Wave 0) and first post-COVID-19 time point (Wave 1). Data collection for the second time point, Wave 2, is currently ongoing (January-July 2022). Pre-COVID-19 data are from Phase 3 of B-Proact1v, a longitudinal study which has been described in detail elsewhere [9]. This study collected questionnaire and accelerometer data from Year 6 children (aged 10–11 years) and at least one of their parents from 50 schools in and around Bristol, UK between March 2017 and May 2018. We invited the same 50 schools to participate in the Active-6 study, with all Year 6 children and one parent per family eligible to take part. As in Wave 0, we collected both questionnaire and accelerometer data from both child and parent. Of the 30 (60%) schools who responded, 25 (83%) agreed to take part in Wave 1, three refused and two agreed to take part only in Wave 2. Two of the consenting schools had to be rescheduled for Wave 2 due to COVID-19 outbreaks. Data collection in the remaining 23 schools took place in two data collection periods; May—July 2021, in which data collection was fully remote, and September—December 2021 when we were able to go into schools. Both the original and current study received ethical approval from the School of Policy Studies Ethics Committee at the University of Bristol, UK, and parental consent was received for all participants at both time points. In Wave 0, children completed paper questionnaires at school, and parents had a choice of paper or online versions. In Wave 1, because of the pandemic and restrictions to in-person data collection, all questionnaire data were collected online. Full details of data collection procedures under different levels of COVID restrictions are provided in the study protocol [25]. A total of 1296 child and parent pairs participated in Wave 0, and 393 in Wave 1 (Fig. 1).

### Accelerometer data

At each time point, participating children and one of their parents wore a waist-worn ActiGraph wGT3X-BT accelerometer (Actigraph LLC; Florida, US). Participants were asked to wear accelerometers during waking hours for five consecutive days in Wave 0, including two weekend days, and for seven consecutive days in Wave 1. Accelerometer data from both Waves were processed using a script written in the R software [26], available from the Open Science Framework [27]. Data between midnight and 6am were excluded and a valid day of data was defined as at least 500 min of data, after excluding intervals of ≥ 60 min of zero counts allowing up to two minutes of any interruptions (consistent with the B-Proact1v study). Analysis of weekday accelerometer data was restricted to participants who provided at least two valid weekdays of data, and weekend accelerometer data to those who provided at least one valid weekend day of data. Activity was recorded in 10 s epochs as sedentary, light intensity or MVPA using Evenson population-specific cut points for children [28], and Troiano cut points for adults [29] (both derived for waist-worn Actigraph accelerometers), and the mean weekday and weekend minutes of MVPA were derived. We also recorded the average number of minutes of accelerometer wear time.

### Covariates

Parents were asked to report their child’s date of birth, gender and the highest education qualification in the household. Index of Multiple Deprivation [30] (IMD) was determined from home postcode (IMD2015 for Wave 0 and IMD2019 for Wave 1), with higher IMD ranks indicating a greater level of deprivation. At school level, we derived school IMD and Income Deprivation Affecting Children Index (IDACI) score [30] based on school postcode, and used published data on school size and percentage of children receiving free school meals [31].

In Wave 0, child height and weight were measured and recorded to the nearest 0.1 cm and 0.1 kg respectively by trained fieldworkers. During the initial part of Wave 1 we were unable to visit schools in person, and so no height and weight measurements were taken in the first ten schools, but these were collected for subsequent schools after ethical approval was gained on 8^th^ October 2021. We did not collect self-report height and weight data as this would not be comparable with Wave 0 measures and is known to be subject to bias in children [32]. Body mass index (BMI) was calculated where possible and converted to age- and sex-specific standard scores (BMI z-score) based on UK reference curves [33]. Parent BMI was calculated from parent-reported height and weight via questionnaire as weight in kilograms divided by height in metres squared.

Parents and children were asked to complete questionnaires at both time points. Children were asked about the frequency (coded from 0 = ‘Never’ to 3 = ‘5 + days per week’) with which they engaged in different forms of activity outside school hours: sport or exercise club at school, sport or exercise club elsewhere, playing outdoors in their neighbourhood, and playing outdoors at home. These were combined in a total activity participation score from 0 to 12, with a higher value indicating a higher frequency of participation in activities outside school [34]. They were also asked about attendance at active extracurricular clubs. In Wave 0, children were asked how they typically travelled to and from school for each day of the week. As there was very little variation in travel mode across the week [35], we used the modal travel mode to represent typical travel, and in Wave 1 asked children directly for the typical travel across the week. For both waves, we created a binary indicator of whether they typically used active (walk, bike, or scooter) or inactive (car, bus or train) modes of travel in either direction.

Parents were asked about the number of hours they and their child typically spent in screen-viewing activities on weekdays and at weekends. In Wave 0, these questions were asked separately about TV, computers, phones/tablets and games consoles, each coded from 0 = ‘None’ to 5 = ‘4 h or more’, and the midpoints summed to calculate total screen viewing. In Wave 1, parents were asked to report total leisure screen-viewing and TV-viewing on hourly scales for ‘Less than 1 h’ up to ‘ > 3 h’ for TV and up to ‘ > 5 h’ for screen-viewing. We converted both measures to binary indicators of total screen viewing ≥ 2 h and TV viewing ≥ 1 h for weekdays and weekends.

### Statistical analysis

The detailed statistical analysis plan [36] for the project was agreed prior to the analysis being conducted. The primary outcome was average daily child weekday MVPA and main explanatory variable was the difference between Wave 0 and Wave 1, captured via an indicator variable. We reported school-level characteristics, pupil demographics (gender, household education, IMD, BMI z-score where available) and descriptive summaries of key variables for the 50 schools at Wave 0 and 23 participating schools at Wave 1.

This is a repeated cross-sectional design, matched on schools. To allow for this cluster design, all comparisons of between the Wave 0 and Wave 1 data were estimated via multilevel models with children nested within schools and school-level random intercepts. This allowed us to include data from schools that participated in Wave 0 but not Wave 1 (27 schools); such schools contribute information about the baseline levels of physical activity. All physical activity and sedentary time models were adjusted for accelerometer wear time, and seasonality using second order harmonic sine/cosine functions [37], and included the main explanatory variable for differences between waves. Continuous outcomes (MVPA, sedentary time, activity score) were modelled using linear models, and discrete outcomes (meeting UK physical activity guidelines, attending active clubs, using active travel, screen-viewing and TV-viewing) used logistic models. In the first data collection period of Wave 1, some COVID-19 restrictions were still in place and data collection protocols differed. An amendment was made to the statistical analysis plan (approved by the Chair of the Study Steering Committee) after interim data had been analysed but before the full dataset was constructed, to include an indicator variable for this period in all models. For transparency, we report the original unadjusted model as a sensitivity analysis. We reported means and 95% CIs of differences in all key child and parent outcomes firstly from this base model and secondly adjusted for confounders known to be associated with physical activity (child models: gender, household education and parent models: age, gender, household education). To determine whether the impact of COVID-19 on MVPA and sedentary time differed by gender and socio-economic position, we repeated the confounder-adjusted models with interaction terms between wave and gender, and wave and household education, as sample sizes were too small to allow subgroup analyses. Model assumptions of normality and homoskedasticity were checked via visual inspection of the residuals, and multicollinearity was assessed via variance inflation factors.

The role of BMI is complex, as it may be acting as a confounder, mediator or both in this analysis. The COVID-19 pandemic has been associated with increases in BMI z-score [38], and increased adiposity has been found to be a causal risk factor for lower physical activity in children [39]. As specified in the statistical analysis plan, since height and weight were not measured in the first ten schools, we imputed missing BMI z-score data under a missing at random assumption, since these data were missing at school level rather than due to individual characteristics. We imputed other missing data at the same time, to include as much information on participants as possible. We used multilevel joint modelling multiple imputation via the package jomo in R [26] that accounted for the clustering within schools, stratified by child gender and using child (age, gender, MVPA and sedentary time), parent (gender, household education and BMI) and school data (average age, gender, MVPA, household education, parent BMI and parent ethnicity for each school) to impute missing data. Fifty imputed datasets were created using a joint multivariate normal model, fitted by Monte Carlo Markov Chains, with a burn-in of 1000 iterations and 1000 iterations between imputation sets (selected via visual inspection of convergence and autocorrelation of the chains). We then used the imputed data in a sensitivity analysis in which MVPA and sedentary time were additionally adjusted for BMI z-score, with results averaged over imputation datasets using Rubin’s rules [40]. Given the level of missing data, we report imputed BMI-adjusted models for PA outcomes only and note that these analyses are considered exploratory.

Several pre-specified sensitivity analyses were conducted. Firstly, to explore any bias due to different underlying populations, we repeated the main analyses restricting data to the subset of schools with data in both waves. We also explored the change in protocol from five days of accelerometer data in Wave 0 to seven in Wave 1 by selecting a random three consecutive weekdays from the Wave 1 data per school (plus the two weekend days) and repeating the main analyses. Finally, as noted above, we compared imputed analyses to complete case analyses, both with and without child BMI z-score. Descriptive summaries were performed in Stata v15 [41] and all multilevel models were run in MLwiN [42] via the Stata command runmlwin.

## Results

The subsample of Wave 1 schools was broadly comparable to Wave 0 schools, in terms of socio-economic indicators (IMD, Income Deprivation Affecting Children Index (IDACI) score, free school meals), geographical distribution (local authority, urban/rural) and pupil demographics (age, gender, ethnicity) at baseline (Table 1). Of the 1024 eligible pupils in the Wave 1 schools, the overall response rate was 38% (*n* = 393), lower than in Wave 0 (63%), but when data collection was possible in person this rose to 48%. Parent and child demographics between the two Waves were broadly similar (Tables 1 and 2), with both participating schools and participants in Wave 1 slightly more likely to come from households with higher education qualifications and in less deprived areas. Children in Wave 1 had higher BMI z-scores (0.38 in Wave 1, compared to 0.35 in Wave 0), although due to data collection restrictions, height and weight were collected in only 61% of children in Wave 1. Missing data ranged between 3–18% for parent characteristics and 11–25% for accelerometer data, with more valid parent data in Wave 1 but similar levels of missingness for accelerometer data (Table S1).

**Table 1:** Comparison of school-level characteristics for participating schools in Wave 0 and Wave 1

	Wave 0 (Pre-COVID-19)		Wave 1	
	*n* = 50		*n* = 23	
School characteristics in 2021				
Urban: N (%)	45	(90%)	19	(83%)
Local Authority N (%)				
Bath & North East Somerset	4	(8%)	3	(13%)
Bristol	14	(28%)	6	(26%)
North Somerset	12	(24%)	6	(26%)
South Gloucester	20	(40%)	8	(35%)
School size: median (IQR)	231	(141)	236	(184)
School IMD score in 2015^a^: mean (SD)	16.7	(15.3)	13.7	(10.2)
School IDACI: mean (SD)	0.12	(0.11)	0.10	(0.08)
Free school meals %: median (IQR)	11.3%	(11.1%)	10.4%	(12.2%)
Average baseline pupil characteristics per school				
Average child age: mean (SD)	10.9	(0.2)	10.9	(0.2)
Average % girls: mean (SD)	53%	(11%)	52%	(8%)
Average % white British ethnicity: mean (SD)	85%	(13%)	88%	(7%)
Average z-BMI: mean (SD)	0.40	(0.31)	0.29	(0.26)
Average % degree + household education: mean (SD)	53%	(21%)	60%	(16%)
Average pupil IMD: mean (SD)	16.8	(11.5)	14.7	(9.1)
Average weekday min of MVPA: mean (SD)	57.9	(8.9)	60.0	(6.2)
Average weekend min of MVPA: mean (SD)	52.1	(10.2)	52.7	(9.0)

^a^IMD score: lower scores indicate higher deprivation

Wave 0: Mar 2017-Jul 2018; Wave 1: May 2021-Dec 2021

*IMD* Index of Multiple Deprivation, *IDACI* Income Deprivation Affecting Children Index, *IQR* inter-quartile range, *SD* standard deviation, *z-BMI* Body Mass Index standardised z-score, *MVPA* moderate-to-vigorous physical activity

**Table 2:** Comparison of key pupil demographics at Wave 0 and Wave 1

	Wave 0 (Pre-COVID-19)		Wave 1		
	*n* = 1296		*n* = 397		
Child age: mean (SD)	11.0	(0.4)		10.8	(0.5)
Child gender: N (%)					
Male	616	(48%)		193	(50%)
Female	680	(52%)		189	(49%)
Other				2	(1%)
Child z-BMI: mean (SD)	0.35	(1.16)		0.38^a^	(1.24)
Parent age:					
< 35 yrs	106	(10%)		37	(10%)
35–39 yrs	142	(13%)		79	(21%)
40–44 yrs	414	(39%)		136	(35%)
45–49 yrs	273	(26%)		94	(24%)
50 + yrs	128	(12%)		39	(10%)
Parent gender: N (%)					
Male	294	(27%)		91	(24%)
Female	794	(73%)		295	(76%)
Other				1	(< 1%)
Parent ethnicity: N (%)					
White British	944	(87%)		305	(86%)
White other	57	(5%)		23	(7%)
Black/African/Caribbean/Black British	16	(1%)		4	(1%)
Asian/Asian British	40	(4%)		16	(5%)
Mixed	9	(1%)		4	(1%)
Other	17	(2%)		4	(1%)
Household education^c^: N (%)					
Up to GCSE/equivalent	243	(20%)		43	(11%)
A level/equivalent	312	(26%)		93	(24%)
Degree/HND/equivalent	435	(37%)		165	(43%)
Higher degree	201	(17%)		86	(22%)
IMD rank^b^: median (IQR)	22,914	(15,839)		25,523	(11,342)

^a^z-BMI only collected on a subset of 241 children in Wave 1

^b^IMD rank uses 2015 index for Wave 0 and 2019 index for Wave 1, with lower ranks indicating higher deprivation. IMD measures relative deprivation and so ranks can be compared

^c^GCSE = qualification at age 16; A level = qualification at age 18

Wave 0: Mar 2017-Jul 2018; Wave 1: May 2021-Dec 2021

*IMD* Index of Multiple Deprivation, *IQR* inter-quartile range, *SD* standard deviation, *z-BMI* Body Mass Index standardised z-score, *GCSE* General Certificate of Education (qualification at age 16), *HND* Higher National Diploma

**Fig. 2:**
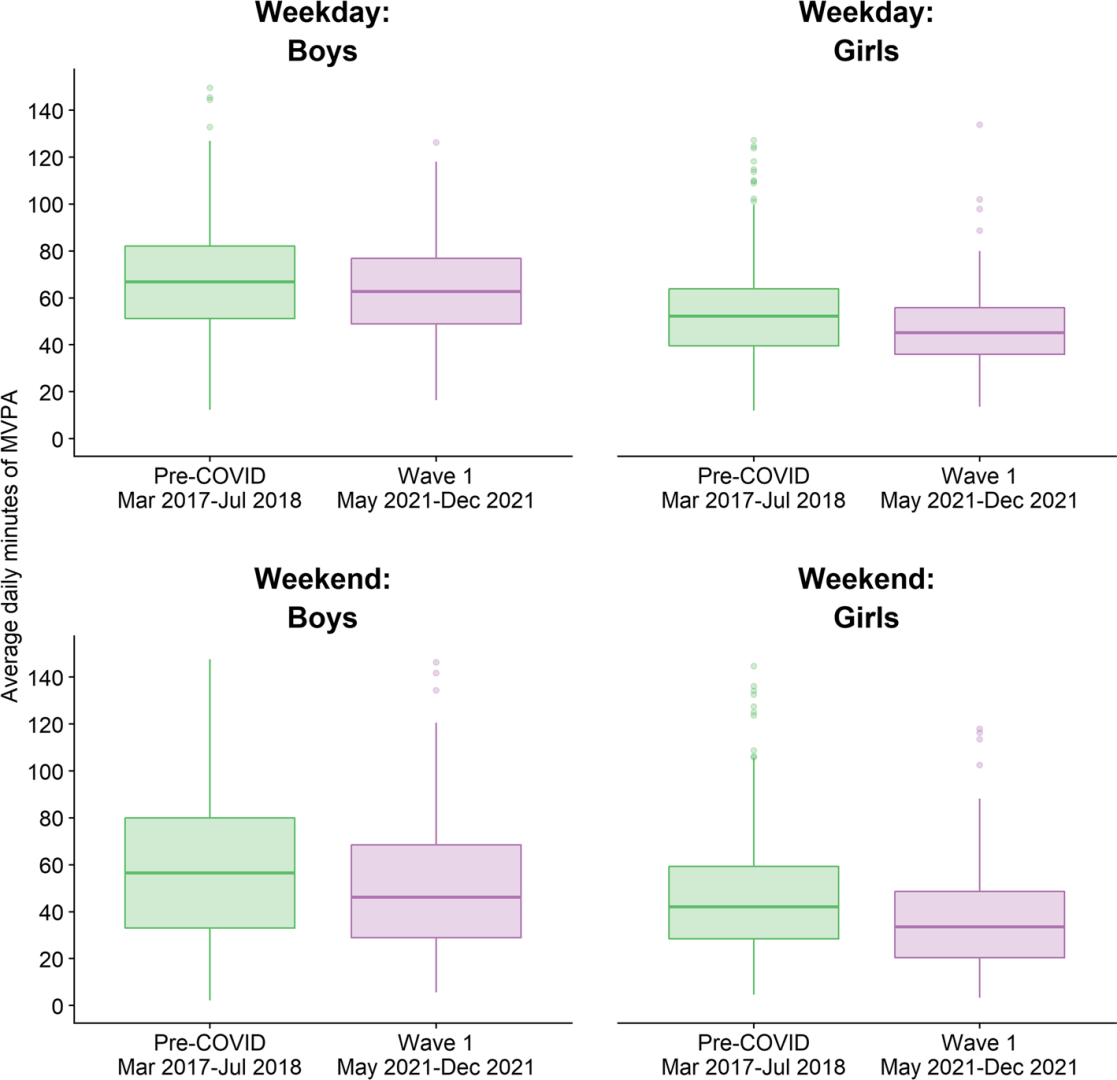
Boxplot of children’s weekday and weekend moderate-to-vigorous physical activity (MVPA) before and after the COVID-19 pandemic by gender (raw data) showing median (middle line), 25^th^ and 75^th^ percentiles (box) and values 1.5 × inter-quartile range (whiskers) from the box

Figure 2 shows the raw data for children’s weekday and weekend MVPA before and after the COVID-19 pandemic by gender. Note that these data and the estimates given in Table 3 are unadjusted for seasonality, accelerometer wear time or study design, and thus we make no assumptions about these, in line with the statistical analysis plan [36] as approved by the Study Steering Committee. Children’s weekday MVPA was lower in Wave 1 than in 2018, at 55.9 min (95% CI: 52.1 to 59.7) and 60.3 min (95% CI: 57.2 to 63.4) respectively. Weekend MVPA was also lower in Wave 1 (45.7 min (95% CI: 41.8 to 49.6) compared to 53.4 min (95% CI: 49.8 to 57.1) in Wave 0), and we saw a reduction in the percentage of children meeting CMO guidelines from 40 to 36% (Table 3). Sedentary time was higher, at 488.4 min on weekdays and 452.5 min at weekends in Wave 1, compared to 476.8 min and 437.1 min in Wave 0 respectively. These patterns were consistent across gender and highest household education. By contrast, parents’ MVPA was similar between Wave 1 and Wave 0, with 82% to 85% meeting CMO guidelines in Wave 0 and Wave 1 respectively (Table 4).

**Table 3:** Estimates of child physical activity for Wave 0 and Wave 1, by gender and household education

	Wave 0 (Pre-COVID-19)		Wave 1	
	Estimate	95% CI	Estimate	95% CI
All children				
Mean weekday MVPA (min/day)	60.3	(57.2, 63.4)	55.9	(52.1, 59.7)
Mean weekday light physical activity (min/day)	209.4	(204.8, 213.9)	198.1	(190.9, 205.3)
Mean weekday sedentary (min/day)	476.8	(470.1, 483.6)	488.4	(477.7, 499.0)
Mean weekend MVPA (min/day)	53.4	(49.8, 57.1)	45.7	(41.8, 49.6)
Mean weekend light physical activity (min/day)	195.2	(190.0, 200.5)	185.4	(179.8, 190.9)
Mean weekend sedentary (min/day)	437.1	(429.3, 444.9)	452.5	(442.6, 462.4)
% meeting UK PA guidelines	40%	(34%, 46%)	36%	(29%, 44%)
Child gender				
Boys				
Mean weekday MVPA (min/day)	68.4	(64.6, 72.2)	64.3	(60.6, 67.9)
Mean weekday light physical activity (min/day)	212.0	(206.7, 271.2)	206.4	(198.1, 214.7)
Mean weekday sedentary (min/day)	466.1	(456.6, 475.7)	478.7	(466.1, 491.4)
Mean weekend MVPA (min/day)	61.1	(56.0, 66.2)	52.6	(48.3, 56.8)
Mean weekend light physical activity (min/day)	193.1	(187.1, 199.1)	190.2	(180.7, 199.7)
Mean weekend sedentary (min/day)	444.3	(432.8, 455.8)	449.6	(434.6, 464.5)
% meeting UK PA guidelines	55%	(48%, 61%)	52%	(44%, 60%)
Girls				
Mean weekday MVPA (min/day)	53.4	(50.3, 56.4)	47.3	(43.1, 51.5)
Mean weekday light physical activity (min/day)	207.1	(201.7, 212.5)	189.7	(182.2, 197.2)
Mean weekday sedentary (min/day)	485.9	(479.2, 492.6)	498.1	(485.9, 510.3)
Mean weekend MVPA (min/day)	46.7	(43.4, 50.1)	38.2	(33.2, 43.2)
Mean weekend light physical activity (min/day)	197.1	(190.5, 203.6)	190.0	(175.1, 186.9)
Mean weekend sedentary (min/day)	430.9	(422.31, 439.5)	455.0	(445.9, 466.1)
% meeting UK PA guidelines	28%	(22%, 34%)	20%	(12%, 27%)
Household education				
Up to A level or equivalent^a^				
Mean weekday MVPA (min/day)	59.8	(56.3, 63.3)	55.0	(50.3, 59.7)
Mean weekday light physical activity (min/day)	211.8	(207.0, 216.7)	200.3	(190.1, 210.5)
Mean weekday sedentary (min/day)	478.3	(469.9, 486.7)	474.5	(460.8, 488.2)
Mean weekend MVPA (min/day)	52.5	(47.8, 57.2)	42.3	(36.0, 48.7)
Mean weekend light physical activity (min/day)	198.5	(191.6, 205.4)	184.7	(176.3, 193.0)
Mean weekend sedentary (min/day)	434.6	(424.1, 445.0)	441.8	(426.6, 457.0)
% meeting UK PA guidelines	39%	(33%, 46%)	39%	(30%, 49%)
Degree equivalent or higher				
Mean weekday MVPA (min/day)	60.5	(57.0, 64.1)	56.4	(52.2, 60.5)
Mean weekday light physical activity (min/day)	206.4	(200.9, 212.0)	196.4	(188.5, 204.2)
Mean weekday sedentary (min/day)	477.4	(469.4, 485.3)	496.6	(485.4, 507.8)
Mean weekend MVPA (min/day)	54.4	(50.2, 58.6)	46.8	(42.7, 509.8)
Mean weekend light physical activity (min/day)	191.5	(185.6, 197.3)	184.1	(177.8, 190.5)
Mean weekend sedentary (min/day)	439.7	(430.1, 449.3)	458.1	(447.5, 468.7)
% meeting UK PA guidelines	41%	(33%, 48%)	35%	(26%, 43%)

Standard errors are adjusted for clustering, but estimates are not adjusted for wear time or seasonality

^a^A level = qualification at age 18

Wave 0: Mar 2017-Jul 2018; Wave 1: May 2021-Dec 2021

*CI* confidence interval, *MVPA* moderate-to-vigorous physical activity, *PA* physical activity

**Table 4:** Estimates of parent physical activity for Wave 0 and Wave 1

	Wave 0 (Pre-COVID-19)		Wave 1	
	Estimate	95% CI	Estimate	95% CI
All parents				
Mean weekday MVPA (min/day)	54.6	(52.2, 57.0)	55.6	(51.7, 59.5)
Mean weekday light physical activity (min/day)	198.5	(193.9, 203.1)	190.8	(184.7, 196.8)
Mean weekday sedentary (min/day)	542.3	(535.4, 549.2)	521.6	(512.0, 531.3)
Mean weekend MVPA (min/day)	46.4	(43.2, 49.6)	49.0	(44.0, 54.0)
Mean weekend light physical activity (min/day)	191.6	(186.8, 196.5)	190.6	(183.5, 197.8)
Mean weekend sedentary (min/day)	486.5	(480.4, 492.6)	486.8	(475.0, 498.6)
% achieving 150 + min/wk	82%	(79%, 86%)	85%	(80%, 89%)

Standard errors are adjusted for clustering, but estimates are not adjusted for wear time or seasonality

Wave 0: Mar 2017-Jul 2018; Wave 1: May 2021-Dec 2021

*CI* confidence interval, *MVPA* moderate-to-vigorous physical activity

**Fig. 3:**
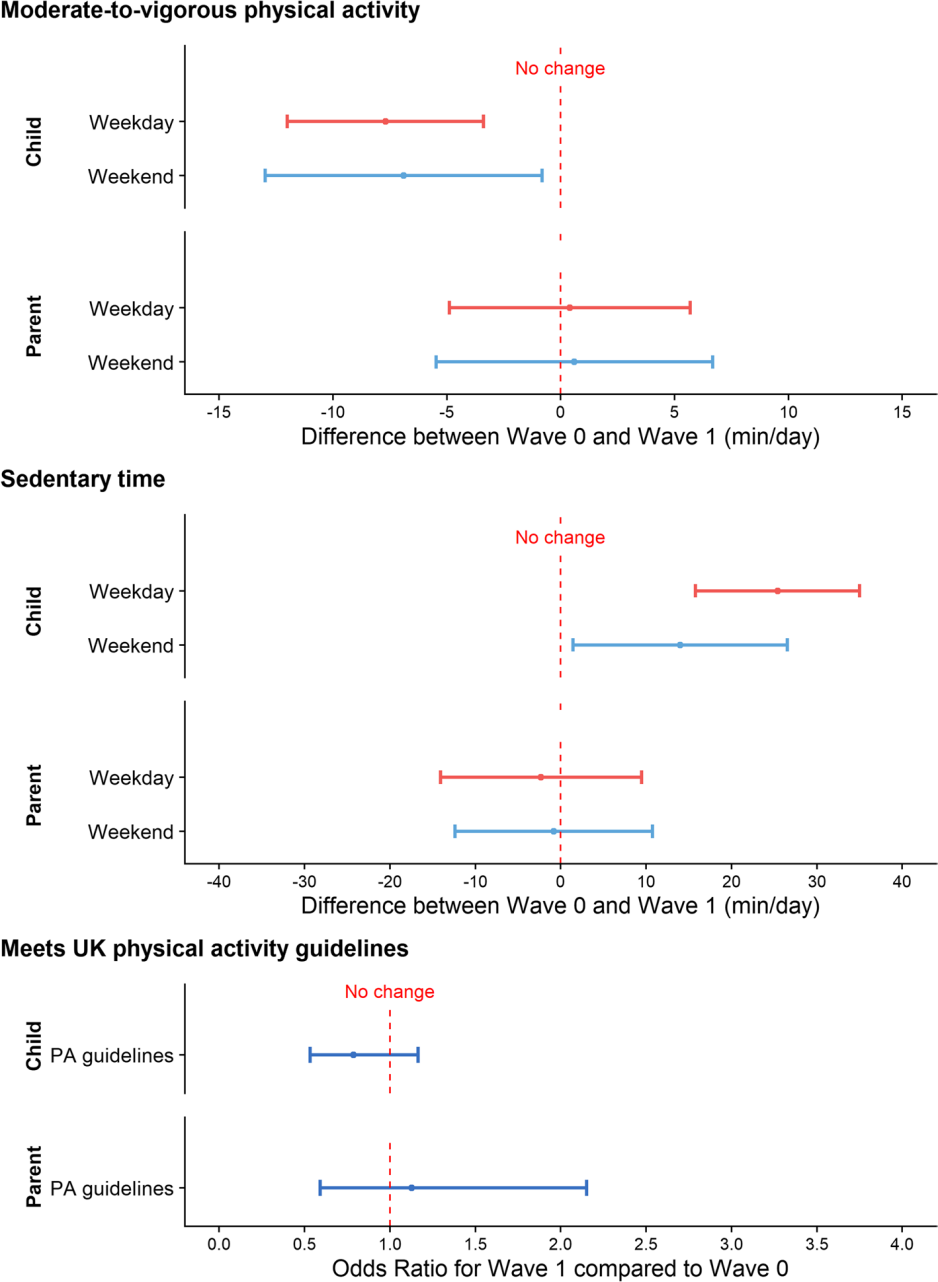
Model estimates and 95% confidence intervals for the difference in physical activity summaries for children and parents between Wave 0 (pre-COVID-19: Mar 2017-Jul 2018) and Wave 1 (May 2021-Dec 2021). Note ‘Weekend’ refers to a weekend day

The estimated difference in children’s weekday MVPA (Table 5) between pre-COVID-19 and Wave 1 was -7.7 min (95% CI: -11.9 to -3.5) after adjusting for gender, age and highest household education. After additionally adjusting for BMI z-score (using multiple imputation for the missing z-BMI) the difference was -6.5 (95% CI: -10.8 to -2.3). There was no evidence of an interaction with gender, household education or BMI z-score. Tables S2 and S3 report estimates of the difference for secondary outcomes for confounder-adjusted models, with estimated differences for physical activity and sedentary time shown in Fig. 3. Children’s weekend MVPA showed a similar difference of -6.9 min (95% CI: -12.9 to -0.9) and increases of 25.4 min (95% CI: 15.8 to 35.0) and 14.0 min (95% CI: 1.5 to 26.5) in sedentary time for weekdays and weekends respectively. After adjusting for BMI z-score, these differences were attenuated slightly by approximately 1 min (Table S4). Parents’ MVPA and sedentary time were similar in Wave 1 compared to pre-COVID-19.

**Table 5:** Modelled difference in child weekday MVPA between Wave 0 and Wave 1

	Difference between Wave 0 and Wave 1			Interaction effect (interaction with Wave)		
	Estimate	95% CI	*p*-value^a^	Estimate	95% CI	*p*-value^b^
Model 1: unadjusted	-6.7	(-11.0, -2.3)	0.003			
Model 2: adjusted	-7.7	(-11.9, -3.5)	< 0.001			
Model 3: adjusted + z-BMI^c^	-6.5	(-10.8, -2.3)	0.002			
Model 4: gender interaction	-7.9	(-12.7, -3.2)	0.001	0.6	(-4.2, 5.3)	0.818
Model 5: education interaction	-7.5	(-12.2, -2.8)	0.002	-0.4	(-5.4, 4.6)	0.876
Model 6: z-BMI interaction^c^	-6.2	(-10.5, -1.9)	0.005	-0.6	(-2.9, 1.6)	0.579

All models are adjusted for accelerometer wear time, seasonality and remote data collection

Models 2 & 3 also control for gender, age and household education

^a^*p*-value for a test for a difference between Wave 0 and Wave 1

^b^*p*-value for a test for an interaction effect

^c^z-BMI models use multiple-imputed data: see text for details

Wave 0: Mar 2017-Jul 2018; Wave 1: May 2021-Dec 2021

*CI* confidence interval, *MVPA* moderate-to-vigorous physical activity, *CMO* Chief Medical Officer, *PA* physical activity, *z-BMI* Body Mass Index standardised z-score

There was negligible difference in participation in active after-school clubs or active travel (Table S3). Children’s overall activity score (which captures a mix of structured clubs and unstructured active play across the week) was lower in Wave 1, equating to roughly 1–1.5 fewer days per week engaged in these types of activities (Table S2). There were differences in screen viewing for both children and parents. The proportion of children and parents who engaged in more than 2 h of leisure screen-viewing was lower in Wave 1 but the proportion who engaged in more than 1 h of TV-viewing was higher. (Tables S2 and S3).

Model checking showed no issues with normality assumptions or multicollinearity. Sensitivity analyses showed that restricting analysis to just those schools that took part in both waves had only small impact on the results, increasing the pre/post COVID-19 difference by around 2 min (Table S5). There was a notable difference between the first data collection period, when data collection was undertaken remotely and school-specific COVID-19 measures were in place, and the later data collection period, with the initial period showing an increase in weekday MVPA of 11.0 min (95% CI: 3.3 to 18.7) compared to Wave 0, in contrast to the decrease seen subsequently. The models reported above adjusted for these differences; when no adjustment is included, the overall pre/post COVID-19 difference in weekday MVPA was smaller at -3.8 min (95% CI: -7.1 to -0.5) (Table S5). Differences in physical activity and sedentary time estimates between the B-Proact1v protocol (summaries based on three weekdays) and Active-6 (summaries based on five weekdays) were negligible (Table S6). There were negligible differences between complete case and imputed models that exclude BMI z-score (7.7 min/day and 7.2 min/day lower children’s weekday MVPA post COVID-19 respectively; Table S7).

## Discussion

The data presented in this paper suggest that children’s MVPA was lower post-COVID-19 by around 7–8 min per day on both weekdays and weekends when compared to data collected in the same schools, in the same way, prior to the COVID-19 pandemic. The data have also shown that average daily sedentary time was higher for children by nearly half an hour during the week and quarter of an hour at weekends. There was no evidence, however, that these differences were associated with gender or SEP. The reason for this reduction is unclear. Although the two waves were conducted 3 years apart, we are not aware of any study that has reported population change previously over such a short period, so we believe that the COVID-19 pandemic, associated restrictions and/or behaviour change in response are at least in part responsible. More detailed exploration of possible reasons is beyond the scope of the current paper, but we hope to explore this in more depth in qualitative analysis of the Active-6 project. The decrease in MVPA reported in this study is slightly lower than the decreases of 10–17 min found in the only other studies [15–17] we identified using device-measured MVPA, all outside the UK. It is important to note, however, that these studies had much smaller sample sizes and the data were collected during the first lockdown period in 2020, during school closures. Our data collection was predominantly in Autumn 2021 when lockdown restrictions had mostly been removed. The key message from this study is therefore that the initial detrimental impact of the pandemic on children’s physical activity appears to continue beyond the period of actual restrictions. This is supported by the findings of the ALCYPS survey, which found a decrease in the number of active children in the initial lockdown May–July 2020 compared to the previous year, but no subsequent recovery [18]. This is concerning as children’s MVPA declines with age at a rate of on average 2.2 min per year [9], so a reduction of 8 min due to the pandemic is potentially accelerating children three years along that age-related decline. Evidence around longer-term trajectories is limited, and so it is difficult to quantify the potential impact of a reduction in MVPA of this size. However, previous studies [43, 44] have reported inverse associations between physical activity and cardiometabolic risk factors, such as insulin, glucose, triglycerides and HDL-cholesterol, which would potentially suggest that some children may be at increased cardiometabolic risk after the pandemic.

Previous studies have reported large decreases in adult physical activity during initial lockdowns, with limited evidence suggesting that while activity levels increase as restrictions are eased, for many they do not return to pre-COVID-19 levels [45–47]. However, the parents in our study had similar levels of MVPA and sedentary time post-COVID-19 when compared to parents recruited from the same schools three years earlier. It should be noted, however, that these are parents of Year 6 children who were mainly female, aged 35–49 and with higher education levels than the general population, and so these findings may not be applicable to all adults. As the impact of lockdowns on physical activity has differed by age, gender and SEP, it may be that recovery will differ as well. More research using device-based measures of physical activity is needed to explore whether these results hold more widely, and in particular to identify groups who may need additional support.

The role of BMI is complex, and further complicated in our study by COVID-19-related data collection issues. A UK longitudinal study found an increase in child BMI over the period of the pandemic [38], but as there is some evidence that children with higher BMI are less active (although the direction of association is not clear) [9, 39], it is difficult to separate the effect of COVID-19 restrictions on physical activity from indirect effects on BMI. When adjusting for BMI z-score, the difference in MVPA pre and post COVID-19 was slightly smaller but still evident, suggesting that the pandemic has impacted both BMI and physical activity. As noted above it is important to highlight that we had a large amount of missing BMI data and had to impute data, so extra caution is required when interpreting the results of analyses that included z-BMI.

We found no evidence of a difference in participation in extra-curricular clubs taking place at the school, but there was a reduction in the activity score, which includes community clubs and unstructured play, amounting to approximately one day fewer per week. Participation in these activities differs by gender and socioeconomic position [34, 35], and so further investigation is warranted into whether the type of activities engaged in and where they take place has changed since the pandemic, especially for key groups who are typically less active. Leisure screen-viewing was lower for both children and parents on both weekdays and weekends, although care should be taken in making these comparisons as the measures were somewhat different across the two waves. While this contrasts with patterns seen elsewhere during lockdowns [15, 16], other studies typically did not separate leisure and school screen viewing. In our study a third of parents reported post-COVID-19 restrictions that their child spent more than an hour per day engaged in screen viewing for schoolwork. TV viewing (including on-demand TV) was substantially higher in Wave 1 for both adults and children than during Wave 0, with the odds of children watching more than one hour of TV two to three times higher. This may reflect a change in screen-viewing patterns, with an increase in family TV time and screen-viewing for schoolwork but reduced use of phones and games consoles. Ongoing qualitative work as part of the Active-6 project will explore changes in screen-viewing in more depth.

In this paper we have focused on differences in physical activity and sedentary time between pre-COVID-19 and Autumn 2021, adjusted for contextual differences in the initial data collection period (May–July 2021). However, the data suggest that children’s PA levels were higher than pre-COVID-19 by around 10 min during that period, compared to the reduction seen subsequently. Some of this may be due to differential recruitment with both participating schools and pupils in that time likely to be more active, with lower BMI z-score and from higher educated and less deprived households. However, it is also likely that the post-lockdown impact of the COVID-19 pandemic is not constant but changing over time, in response to local risk assessment and disruptions as well as changing behaviour. Understanding and monitoring the longer-term impacts is crucial, in particular how these change over time, and is the focus of continued data collection in the Active-6 project.

### Strengths and limitations

It is important to recognise several limitations. We experienced poorer response rates due to COVID-19 (including fewer participating schools), changes in data collection protocols, differing school risk assessments and disruption due to COVID-19 within schools, all of which affect the amount of data that we were able to collect. This was expected given the challenges of conducting school-based research during a pandemic. This is a repeated cross-sectional study, which is appropriate to investigate physical activity and sedentary time of children and their parents before and after the COVID-19 restrictions, but lack of longitudinal data means we do not know how active children were before, or if this affected participation. While the participating schools were demographically similar to the original Wave 0 cohort, schools with pupils who were previously more active were more likely to take part. We are therefore unable to determine if the pandemic has differentially affected the most or the least active children. To facilitate comparison with Wave 0 data and the wider field, we processed the accelerometer data using widely used cut-points that were developed for earlier versions of the Actigraph accelerometer. It is possible that alternative data processing approaches, including compositional data analysis, may have yielded different results. Finally, as for any analysis covering the period of the COVID-19 pandemic, we cannot rule out that differences are due to changes in physical activity and sedentary levels over time driven by factors other than COVID-19, although the scale of the observed differences and other evidence support our interpretation. However, our study also has several strengths. Despite the difficulty in collecting data on children in schools over the pandemic, we were able to build on our excellent existing relationships with local schools to collect much-needed data on physical activity and sedentary time. Both pre-COVID-19 comparator data and data for the current study were measured contemporaneously by accelerometers, rather than relying on retrospective self-report measures of activity, and collected as far as possible under the same circumstances. Moreover, the data were collected in the same schools, which in the case of this study increased the statistical power of the study and reduced potential for school-level imbalance between pre- and post-COVID-19 measures. We have also concentrated on understanding the longer-term impacts of the pandemic once restrictions are lifted, an area where there is little current evidence, but potentially of great policy relevance.

## Conclusion

Children’s MVPA was lower by 7–8 min per day in 2021 once restrictions were lifted than before the COVID-19 pandemic across gender and SEP, on both weekdays and weekends, equating to a three-year acceleration in otherwise expected age-related decline. Parents’ physical activity and sedentary time were similar to pre-pandemic levels. Previous evidence has shown substantial decreases in children’s physical activity during lockdowns associated with the COVID-19 pandemic, and our results suggest that despite easing of restrictions, children’s activity levels have not returned to pre-pandemic levels. There is an urgent need to understand why these changes have occurred and whether they are maintained during the recovery from the COVID-19 pandemic.

## Supplementary Information

**Additional file1: Table S1. **Summary of missing data. **Table S2.** Modelled difference in continuouschild and parent outcomes between Wave 0 and Wave 1. **Table S3.** Modelled difference in discretechild and parent outcomes between Wave 0 and Wave 1. **Table S4.** Child physical activity outcomes,mediated by BMI (using multiple imputation). **Table S5.** Children’s weekday MVPA:sensitivity analysis for schools and differences in data collection/COVIDrestrictions. **Table S6.** Comparison of Wave 1accelerometer summaries for three weekdays (BProact1v protocol) and fiveweekdays (Active 6 protocol). **Table S7.** Comparison of complete case andimputed data for children’s weekday MVPA models including BMI 

## Data Availability

As the Active-6 project is still ongoing, data are not currently available. At the end of the project data will be made available in the University of Bristol’s data repository (https://data.bris.ac.uk/data/).

## Abbreviations

ALCYPS: Active Lives Children and Young People Survey
BMI: Body Mass Index
CMO: Chief Medical Officers
IDACI: Income Deprivation Affecting Children Index
IMD: Index of Multiple Deprivation
MVPA: Moderate-to-vigorous physical activity
SEP: Socioeconomic position
z-BMI: BMI standardised z-score
