# Supplementary material for: Accelerometer-measured physical activity and sedentary time among children and their parents in the UK before and after COVID-19 lockdowns: a natural experiment
Source: Int J Behav Nutr Phys Act. 2022 May 16;19:51. doi: 10.1186/s12966-022-01290-4 (PMC9107948; doi:10.1186/s12966-022-01290-4)
Supplement: Supplementary file 1 — Additional file1: Table S1. Summary of missing data. Table S2. Modelled difference in continuouschild and parent outcomes between Wave 0 and Wave 1. Table S3. Modelled difference in discretechild and parent outcomes between Wave 0 and Wave 1. Table S4. Child physical activity outcomes,mediated by BMI (using multiple imputation). Table S5. Children’s weekday MVPA:sensitivity analysis for schools and differences in data collection/COVIDrestrictions. Table S6. Comparison of Wave 1accelerometer summaries for three weekdays (BProact1v protocol) and fiveweekdays (Active 6 protocol). Table S7. Comparison of complete case andimputed data for children’s weekday MVPA models including BMI [file 12966_2022_1290_MOESM1_ESM.docx]

# Supplementary Material:

**Accelerometer-measured physical activity and sedentary behaviour among children and their parents in the UK before and after COVID-19 lockdowns: a natural experiment**

Ruth Salway, Charlie Foster, Frank de Vocht, Jo Williams, Byron Tibbitts, Lydia Emm-Collison, Danielle House, Katie Breheny, Tom Reid, Robert Walker, Sarah Churchward, William Hollingworth and Russell Jago

Table S1: Summary of missing data

Table S2: Modelled difference in continuous child and parent outcomes between Wave 0 and Wave 1

Table S3: Modelled difference in discrete child and parent outcomes between Wave 0 and Wave 1

Table S4: Child physical activity outcomes, mediated by BMI (using multiple imputation)

Table S5: Children’s weekday MVPA: sensitivity analysis for schools and differences in data collection/COVID restrictions

Table S6: Comparison of Wave 1 accelerometer summaries for three weekdays (BProact1v protocol) and five weekdays (Active 6 protocol)

Table S7: Comparison of complete case and imputed data for children’s weekday MVPA models including BMI

**Table S1: Summary of missing data**

|  |  | Wave 0  (Pre-COVID-19)  *n*=1296 | | Wave 1  *n*=397 | |
| --- | --- | --- | --- | --- | --- |
|  |  | missing | | missing | |
|  | | N | % | N | % |
| Child | |  |  |  |  |
| Age | | 0 | 0% | 0 | 0% |
| Gender | | 0 | 0% | 4 | 1% |
| z-BMI | | 11 | 1% | 148 | 38% |
| Weekday accelerometer data | | 173 | 13% | 39 | 10% |
| Weekend accelerometer data | | 332 | 26% | 102 | 26% |
| Parent | |  |  |  |  |
| Age | | 233 | 18% | 6 | 2% |
| Gender | | 208 | 16% | 7 | 2% |
| Ethnicity | | 213 | 16% | 32 | 8% |
| Weekday accelerometer data | | 270 | 21% | 61 | 16% |
| Weekend accelerometer data | | 313 | 24% | 96 | 24% |
| Household | |  |  |  |  |
| Household education | | 105 | 8% | 6 | 2% |
| IMD score: mean (SD) | | 45 | 3% | 5 | 1% |

Wave 0: Mar 2017-Jul 2018; Wave 1: May 2021-Dec 2021

IMD=Index of Multiple Deprivation; z-BMI=Body Mass Index standardised z-score

**Table S2: Modelled difference in continuous child and parent outcomes between Wave 0 and Wave 1**

|  | Difference between Wave 0 (pre-COVID-19) and Wave 1 | | |
| --- | --- | --- | --- |
|  | Estimate | 95% CI | p-value^1^ |
| CHILD |  |  |  |
| Weekday MVPA^2^ (min/day) | -7.7 | (-11.9, -3.5) | <0.0005 |
| Weekend MVPA^2^ (min/day) | -6.9 | (-12.9, -0.9) | 0.024 |
| Weekday light intensity^2^ (min/day) | -17.7 | (-25.0, -10.4) | <0.0005 |
| Weekend light intensity^2^ (min/day) | -6.7 | (-14.9, 1.4) | 0.105 |
| Weekday sedentary time^2^ (min/day) | 25.4 | (15.8, 35.0) | <0.0005 |
| Weekend sedentary time^2^ (min/day) | 14.0 | (1.5, 26.5) | 0.028 |
| Overall activity score | -1.05 | (-1.46, -0.63) | <0.0005 |
| PARENT |  |  |  |
| Weekday MVPA^2^ (min/day) | 0.4 | (-4.9, 5.8) | 0.880 |
| Weekend MVPA^2^ (min/day) | 0.6 | (-5.5, 6.7) | 0.849 |
| Weekday light intensity^2^ (min/day) | 1.6 | (-7.6, 10.7) | 0.734 |
| Weekend light intensity^2^ (min/day) | -0.3 | (-9.0, 8.4) | 0.942 |
| Weekday sedentary time^2^ (min/day) | -2.3 | (-14.1, 9.5) | 0.705 |
| Weekend sedentary time^2^ (min/day) | -0.8 | (-12.3, 10.6) | 0.887 |

All models are adjusted for accelerometer wear time, seasonality, gender, age and household education.

^1^ p-value for a test for a difference between Wave 0 and Wave 1

^2^ models additionally adjusted for accelerometer wear time.

Wave 0: Mar 2017-Jul 2018; Wave 1: May 2021-Dec 2021

CI=confidence interval; MVPA=moderate-to-vigorous physical activity

**Table S3: Modelled difference in discrete child and parent outcomes between Wave 0**

**and Wave 1**

|  | Wave 1 compared to Wave 0 (pre-COVID-19) | | | |
| --- | --- | --- | --- | --- |
|  | | OR | 95% CI | p-value^1^ |
| Child |  | |  |  |
| % meeting guidelines^2^ | 0.78 | | (0.48, 1.29) | 0.336 |
| % attending active clubs | 1.30 | | (0.84, 2.02) | 0.236 |
| % active travel to or from school | 0.93 | | (0.59, 1.48) | 0.772 |
| Weekday screen viewing^3^ ≥2h | 0.38 | | (0.25, 0.58) | <0.0005 |
| Weekend screen viewing ≥2h | 0.63 | | (0.39, 1.04) | 0.069 |
| Weekday TV viewing ≥1h | 3.07 | | (2.07, 4.55) | <0.0005 |
| Weekend TV viewing ≥1h | 2.68 | | (1.63, 4.41) | <0.0005 |
| Parent |  | |  |  |
| % meeting guidelines^1^ | 1.13 | | (0.64, 2.00) | 0.677 |
| Weekday screen viewing ≥2h | 0.19 | | (0.13, 0.29) | <0.0005 |
| Weekend screen viewing ≥2h | 0.24 | | (0.15, 0.37) | <0.0005 |
| Weekday TV viewing ≥1h | 2.63 | | (1.71, 4.04) | <0.0005 |
| Weekend TV viewing ≥1h | 1.60 | | (0.93, 2.77) | 0.09 |

All models are adjusted for seasonality, gender, age and household education.

^1^ p-value for a test for a difference between Wave 0 and Wave 1

^2^ models additionally adjusted for accelerometer wear time

^3^ excluding schoolwork

Wave 0: Mar 2017-Jul 2018; Wave 1: May 2021-Dec 2021

OR=Odds ratio; CI=confidence interval; MVPA=moderate-to-vigorous physical activity

**Table S4: Child physical activity outcomes, mediated by z-BMI (using multiple imputation)**

|  | Difference between pre-COVID-19 and Wave 1 (min/day) | | |
| --- | --- | --- | --- |
|  | Estimate | 95% CI | p-value^1^ |
| CHILD |  |  |  |
| Weekday MVPA^2^ | -6.5 | (-10.8, -2.3) | 0.002 |
| Weekend MVPA^2^ | -6.2 | (-12.2, -0.3) | 0.040 |
| Weekday sedentary time^2^ | 23.5 | (14.0, 33.0) | <0.0005 |
| Weekend sedentary time^2^ | 11.7 | (-0.1, 23.4) | 0.052 |

All models are adjusted for accelerometer wear time, seasonality, gender, age, household education and BMI z-score

^1^ p-value for a test for a difference between Wave 0 and Wave 1

^2^ models additionally adjusted for accelerometer wear time.

Wave 0: Mar 2017-Jul 2018; Wave 1: May 2021-Dec 2021

CI=confidence interval; MVPA=moderate-to-vigorous physical activity; z-BMI=Body Mass Index standardised z-score

**Table S5: Children’s weekday MVPA: sensitivity analysis for schools and differences in data collection/COVID restrictions**

|  |  | Difference from Wave 0 (pre-COVID-1) (min/day) | | |
| --- | --- | --- | --- | --- |
|  |  | Estimate | 95% CI | p-value |
| Full model: *n*=1388 | | | | |
|  | Initial Wave 1 period^1^ | 11.0 | (3.3, 18.7) | 0.005 |
|  | Wave 1 difference | -7.7 | (-11.9, -3.5) | <0.0005 |
| Restricted to schools that took part in both waves: *n*=867 | | | | |
|  | Initial Wave 1 period^1^ | 12.0 | (3.8, 20.2) | 0.004 |
|  | Wave 1 difference | -9.8 | (-14.3, -5.3) | <0.0005 |
| No adjustment for initial Wave 1 period^1^: *n*=1388 | | | | |
|  | Wave 1 difference | -3.8 | (-7.1, -0.5) | 0.022 |
| Excluding schools from initial Wave 1 period^1^: *n*=1295 | | | | |
|  | Wave 1 difference | -7.8 | (-12.2, -3.4) | 0.001 |

^1^ initial period of Wave 1 (May 2021-Jul 2021) used remote data collection, and occurred while stricter COVID restrictions were implemented in schools.

Wave 0: Mar 2017-Jul 2018; Wave 1: May 2021-Dec 2021

CI=confidence interval; MVPA=moderate-to-vigorous physical activity; z-BMI=Body Mass Index standardised z-score

**Table S6: Comparison of Wave 1 accelerometer summaries for three weekdays (B-Proact1v protocol) and five weekdays (Active-6 protocol)**

|  | Three weekdays  (BProact1v protocol) | | Five weekdays  (Active 6 protocol) | |
| --- | --- | --- | --- | --- |
|  | Mean | SD | Mean | SD |
| Child weekday MVPA (min/day) | 55.4 | 23.4 | 55.8 | 21.3 |
| Child weekday sedentary (min/day) | 493.0 | 69.9 | 486.3 | 66.7 |
| Parent weekday MVPA (min/day) | 57.3 | 29.2 | 55.4 | 28.0 |
| Parent weekday sedentary (min/day) | 523.3 | 87.4 | 521.3 | 83.8 |

Note: both protocols collect two days of weekend data and so are directly comparable.

Wave 0: Mar 2017-Jul 2018; Wave 1: May 2021-Dec 2021

MVPA=moderate-to-vigorous physical activity; SD=standard deviation

**Table S7: Comparison of complete case and imputed data for children’s weekday MVPA models**

|  | Difference between Wave 0 (pre-COVID-19) and Wave 1 | | | | | |
| --- | --- | --- | --- | --- | --- | --- |
|  | Complete case | | | Imputed | | |
|  | Estimate | 95% CI | p-value | Estimate | 95% CI | p-value |
| Model excluding zBMI | | | | | | |
| Intercept | 94.4 | (60.2, 128.5) |  | 92.2 | (57.6, 126.9) |  |
| Female | -15.3 | (-17.4, -13.2) | <0.0005 | -14.9 | (-16.9, -12.9) | <0.0005 |
| Age | -2.5 | (-5.7, 0.6) | 0.111 | -2.4 | (-5.5, 0.8) | 0.146 |
| Degree-educated | 0.01 | (02.2, 2.2) | 0.990 | 0.1 | (-2.3, 2.4) | 0.961 |
| Initial Wave 1 period^1^ | 11.0 | (3.3, 18.7) | 0.005 | 8.4 | (0.8, 16.1) | 0.030 |
| Wave | -7.7 | (-11.9, -3.5) | <0.0005 | -7.2 | (-11.5, -2.9) | 0.001 |

^1^ initial period of Wave 1 (May 2021-Jul 2021) used remote data collection, and occurred while stricter COVID restrictions were implemented in schools.

Wave 0: Mar 2017-Jul 2018; Wave 1: May 2021-Dec 2021

CI=confidence interval; MVPA=moderate-to-vigorous physical activity; z-BMI=Body Mass Index standardised z-score
